# Somatotype, anthropometric characteristics, body composition, and global flexibility range in artistic gymnasts and sport hoop athletes

**DOI:** 10.1371/journal.pone.0312555

**Published:** 2024-10-24

**Authors:** Mirvana Elizabeth González Macías, Jaime Flores

**Affiliations:** Laboratory Biomechanics, Faculty of Sports Autonomous University of Baja California, Mexicali, BC, México; West Virginia University, UNITED STATES OF AMERICA

## Abstract

The objective of the study was to analyze the somatotype, anthropometric characteristics, body composition, and the global flexibility battery test. A total of 48 athletes of both sexes from Women’s Artistic Gymnastics, Men’s Artistic Gymnastics, and Hoop Sport (mean ± standard deviation, age 12.50 ± 2.67 years, body mass 43.16 ± 11.00 kg, height 150.15 ± 11.91 cm). Anthropometric data were obtained using the ISAK protocol. The somatotype was analyzed using the Heath-Carter method. The results indicate significant differences in fat, bone, and residual mass, as well as in the proportions of endomorphy, mesomorphy, and ectomorphy (p <0.05). The somatocard revealed that most athletes were classified as endomorphic mesomorph or ectomorphic mesomorph, with variations between groups. Positive and negative correlations were identified between the anthropometric variables, somatotype, body composition, and global flexibility. All positions of the global flexibility battery test showed negative correlations with residual mass, indicating that the greater the range of flexibility, the lower the residual mass.

## Introduction

Dr. Aristides Lanier Soto [1], a recognized expert in the field of sports classification, has defined an essential group within the coordination and competitive art sports (ARC). According to his classification, this group includes disciplines such as Rhythmic Gymnastics (RG), Artistic Gymnastics (AG), Aerobic Gymnastics (AEG), and Synchronized Swimming. Recently, some disciplines have been added to this list, such as Hoop Sport (HS) and Cheerleading (CH), which were officially recognized in México in 2019 and registered with CONADE (National Commission of Physical Culture and Sports). As in all sports, these disciplines require the development of conditional abilities such as speed, strength, endurance, and flexibility to achieve high performance.

To achieve excellence in a sport, it is important to consider multiple factors (physiological, psychological, biomechanical, among others) that influence performance [2]. Body composition is significantly related to physical exercise in both sedentary populations and athletes [3]. Anthropometric parameters differ according to the sport and specific positions when it comes to team sports [4], and are an important parameter of control at the health level and optimal performance [5]. The current concept of somatotype does not suggest a permanent physical classification, as it evaluates the phenotype at a specific moment in life and can change during childhood, adolescence, or other stages due to growth, nutrition, training, or diseases [6]. The somatotype is calculated using three basic components: endomorphy (the level of body fat), mesomorphy (muscle mass), and ectomorphy (bone structure and thinness) [7].

Each sport has a well-defined kinanthropometric pattern. Through this pattern, it is possible to know the anthropometric characteristics that a certain athlete should have to achieve sports success [8, 9]. In many sports, successful athletes seem to have a high proportion of mesomorphy, presenting a strong musculoskeletal development [10].

Competitive art sports are appreciated sports; among the aspects that are scored are technical difficulty, artistic value, execution, and cleanliness in movement, likewise, all these are related to the anthropometric structure [11].

Therefore, the purpose of the present study was to analyze body composition and somatotype in relation to the range of global flexibility in artistic gymnastics and sport hoop athletes.

## Materials and methods

The design of this study is non-experimental, descriptive-correlational, and cross-sectional, as outlined by [12].

### Participants

The study involved 48 athletes, of which 36 were females with an average age of 12.7 ± 2.63 years; body mass 42.95 ± 10.25 kg and height 150.35 ± 10.48 cm; and 12 males, with an average 11.67 ± 2.74 years; body mass 43.77 ± 13.51 kg and height 149.57 ± 16.02 cm. The study was conducted in the city of Mexicali, B.C.; with the support of the following institutions; “Eduardo Carmona Valenzuela” Gymnastics Hall of the UABC Sports Center and Petit Studio from February 21 to November 21, 2022.

The parents of the athletes were informed of the purpose and procedures of the study and gave their informed written consent to participate in the study. This study was conducted in accordance with the principles set forth in the Declaration of Helsinki. Project number 149/3281 received approval by the Ethics Committee of the General Coordination of Graduate Studies and Research at the Autonomous University of Baja California conducted the study. Athletes who presented with any osteomuscular pathology in the last six months were excluded.

### Procedure

In this study, general data such as name, age, and gender were recorded. The International Society for the Advancement of Kinanthropometry (ISAK) has proposed a protocol for collecting anthropometric measures. Weight was measured using an OMRON HBF-514C digital scale, height with SECA wall stadiometer graduated in centimeters, and a Slim guide caliper was used for the measurement of skinfolds (subscapular, triceps, supraspinal, calf), Lufkin tape for the circumferences (contracted arm, calf) and Lafayette brand Vernier for the diameters (humerus and femur).

The data obtained to determine the somatotype of the athletes were entered into the Nutrimind software. The somatotype components were calculated using the Heath and Carter method. Once the values of the three components (Endomorphy, Mesomorphy, and Ectomorphy) were determined, the somatotype of each athlete was calculated. The three-dimensional somatotypic attitudinal distance (SAD) and mean somatotypic attitudinal distance (SAM) were determined, according to the procedure described previously [10]. Following Carter’s recommendations [13], it was established that (SAM > 1.0) is a high distance; (SAM = 0.80–0.99), and a reduced distance (SAM < 0.79).

**Fig 1:**
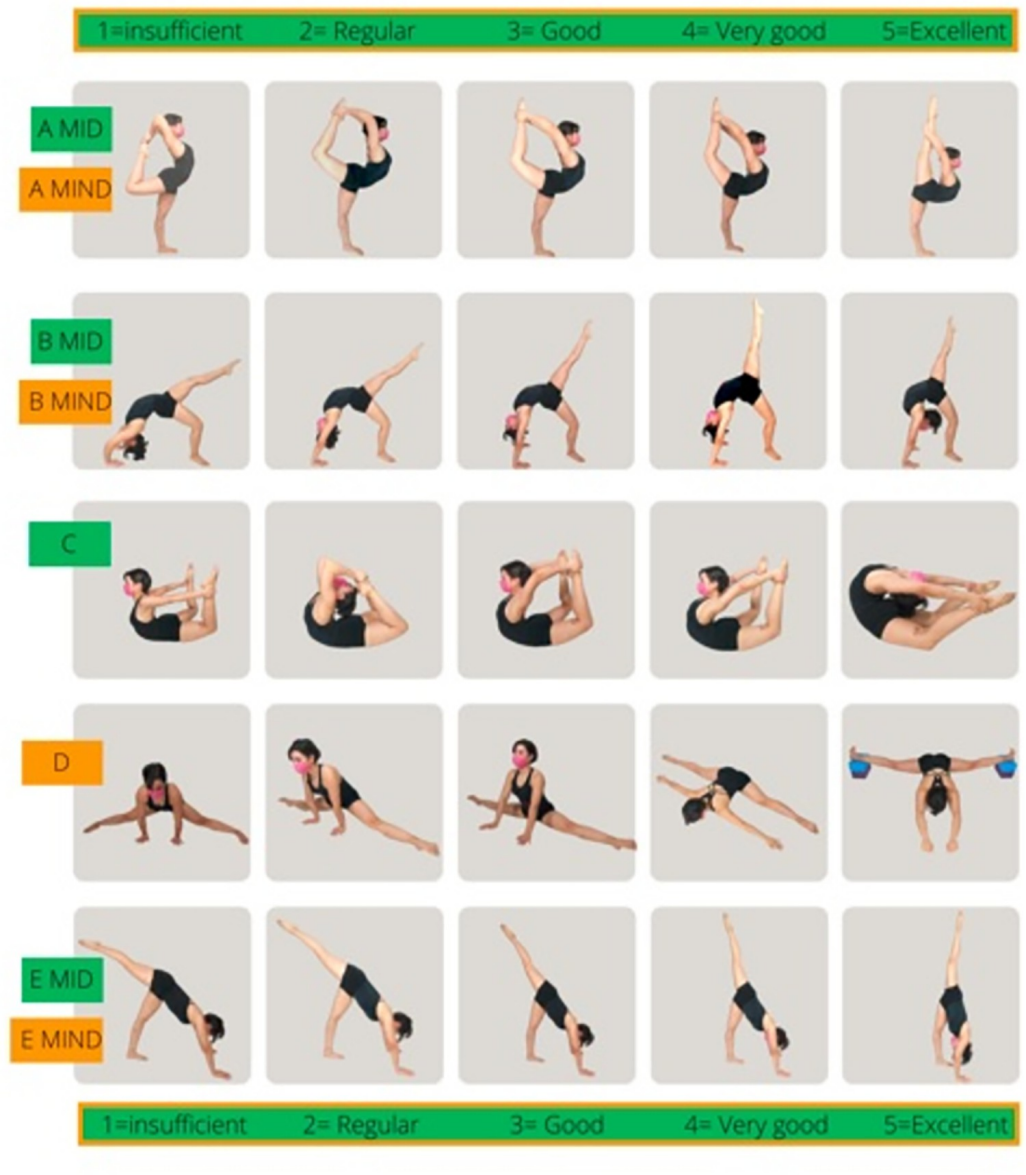
Global flexibility battery test.

The range of flexibility was evaluated with the global flexibility battery test (BFG) proposed by Flores [14]. This consists of the evaluation of 8 specific movements used in competitive art sports: A MID (Element A Dominant Lower Limb), A MIND (Element A Non-Dominant Lower Limb), B MID (Element B Dominant Lower Limb), B MIND (Element A Non-Dominant Lower Limb), C (Element C), D (Element D), E MID (Element E Dominant Lower Limb), and E MIND (Element E Non-Dominant Lower Limb). The proposed exercises originate from the scoring code of women’s artistic gymnastics [15] and the sport hoop code [16] and are routinely performed in training and competition choreographies. Before the test, the athletes perform a specific warm-up to show their ROM (Range of motion). For each movement, six classification values were attributed, referring to the maximum possible amplitudes (Fig 1), an ascending scale from 0 to 5 points was observed, where 0 = Not achieved, 1 = Insufficient, 2 = Regular, 3 = Good, 4 = Very Good, 5 = Excellent.

In the results, only whole numbers are recorded, so movements with intermediate amplitudes between two points on the map are assigned the next lower value. This model was influenced by the approach proposed by Batista, Bobo, Lebre, and Ávila-Carvalho [17].

### Statistical analysis

Statistical analysis was performed using the statistical program SPSS 23.0 (version 23.0 IBM for Windows, SPSS Inc., IL, United States), and two types of analysis were performed: descriptive and inferential. In the descriptive analysis, the Shapiro-Wilk test was applied to evaluate the data distribution. The mean, standard deviation (SD), and Confidence Interval (CI) were determined for all study variables. In the comparisons by discipline, a one-way ANOVA test was performed. Considering all variables had a normal distribution; the Bonferroni post hoc test [18] was applied.

Pearson’s correlation test was used to identify the relationship between the variables evaluated in the athletes. Correlations were defined as very low (0 to 0.1); low (0.1 to 0.3); moderate (0.3 to 0.5), high (0.5 to 0.7), very high (0.7 to 0.9); and almost perfect (0.9 to 1.0) [19]. The significance level for all statistical tests was (p < .05).

## Results

The means, standard deviations, and confidence intervals of the main characteristics of the three study groups are presented. It was observed that the athletes of the men’s Artistic Gymnastics group (MAG) showed statistically significant differences compared to women’s Aerial Hoop Sport (WAHS) and women’s Artistic Gymnastics (WAG). These differences were noted in the following variables: fat mass (p = 0.001), bone mass (p = 0.016), residual mass (p = 0.000), endomorphy (p = 0.004), mesomorphy (p = 0.003), and on the Y-axis Somatotype (p = 0.001), presented in Table 1.

**Table 1:** Anthropometric characteristics, body composition and somatotype of the study participants (n = 48).

Measure	General		WAHS		WAG		MAG		Anova
	n = 48		n = 21		n = 15		n = 12		
	Mean(SD)	CI%	Mean(SD)	CI%	Mean(SD)	CI%	Mean(SD)	CI%	*P*
**Age, (years)**	12.50 ± 2.67	(11.72 13.28)	13.19 ± 2.97	(11.84 14.55)	12.2 ± 2.00	(11.09 13.31)	11.67 ± 2.74	(9.92 13.41)	.257
**Height, (cm)**	150.15 ± 11.91	(146.69 153.61)	150.90 ± 10.22	(146.22 155.55)	149.6 ± 11.15	(139.38 159.74)	149.56 ± 16.02	(139.38 159.74)	.933
**Body Mass, (kg)**	43.16 ± 11.00	(39.96 46.35)	43.62 ± 11.36	(38.44 48.79)	42.03 ± 8.75	(37.18 46.87)	43.77 ± 13.51	(35.18 52.34)	.894
**BMI (*k*/*m*^2^)**	18.81 ± 2.62	(18.05 19.57)	18.84 ± 3.15	(17.40 20.27)	18.62 ± 2.27	(17.36 19.88)	19.02 ± 2.15	(17.65 20.38)	.928
**Fat Mass (kg)**	15.12 ± 5.45	(13.54 16.71)	17.12 ± 5.96	(14.41 19.83)	16.09 ± 4.12	(13.81 18.37)	10.43 ± 2.82	(8.63 12.21)	.001
**Bone Mass, (kg)**	18.51 ± 1.98	(17.94 19.09)	17.87 ± 1.98	(16.97 18.78)	18.34 ± 1.80	(17.34 19.33)	19.86 ± 1.62	(18.83 20.90)	.016
**Residual Mass (kg)**	21.70 ± 1.42	(21.29 22.11)	20.89 ± 0.88	(20.85 20.93)	20.9 ± 0.1	(20.84 20.95)	24.14 ± 0.06	(24.09 24.18)	.000
**Muscle Mass (kg)**	44.65 ± 4.30	(43.40 45.90)	44.10 ± 5.10	(41.78 46.42)	44.65 ± 4.14	(42.35 46.94)	45.60 ± 2.90	(43.75 47.45)	.637
**Endomorphy**	2.75 ± .87	(2.50 3.00)	3.01 ± 1.03	(2.54 3.49)	2.94 ± .56	(2.62 3.25)	2.05 ± .43	(1.78 2.33)	.004
**Mesomorphy**	4.16 ± 1.23	(3.80 4.52)	3.70 ± 1.22	(3.14 4.26)	4.02 ± 1.22	(3.34 4.69)	5.16 ± .62	(4.76 5.56)	.003
**Ectormorphy**	3.03 ± 1.18	(2.69 3.38)	3.12 ± 1.38	(2.49 3.76)	3.09 ± 1.21	(2.42 3.76)	2.80 ± .75	(2.32 3.27)	.736
**X Somatotype**	.28 ± 1.81	(-.24 .80)	.10 ± 2.29	(-.93 1.15)	.15 ± 1.56	(-.71 1.01)	.74 ± .99	(.10 1.37)	.605
**Y Somatotype**	2.54 ± 3.35	(1.57 3.52)	1.26 ± 3.05	(-.12 2.65)	2.00 ± 3.38	(.13 3.87)	5.47 ± 1.87	(4.28 6.66)	.001

**Fig 2:**
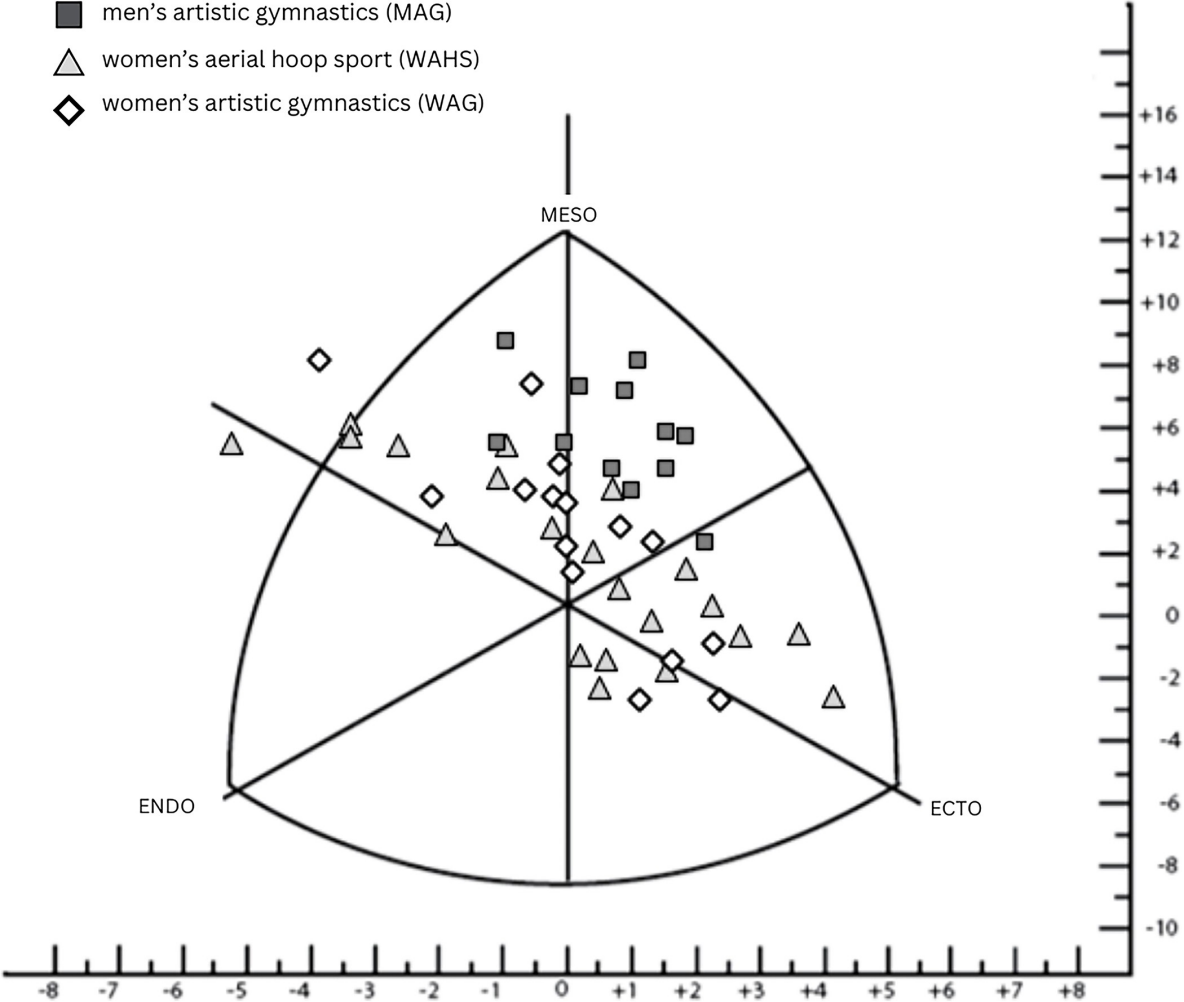
Distribution of somatopoints for men’s artistic gymnastics = MAG, women’s artistic gymnastics = WAG, women’s aerial hoop sport = WAHS.

BMI, body mass index; WAHS, Women’s Aerial Hoop Sport; WAG, Women’s Artistic Gymnastics; MAG, Men’s Artistic Gymnastics; SD, standard deviation; CI, confidence interval.

(Fig 2) presents a somatocard divided into three components; squares represent athletes from men’s Artistic Gymnastics (MAG), diamonds represent women’s Artistic Gymnastics (WAG), and triangles represent athletes from women’s Aerial Hoop Sport (WAHS). Upon analyzing somatocard, it was observed that 29.17% of the athletes were in the endomorphic mesomorph category, this percentage was formed by 12.5% of athletes from (WAHS) and (WAG) and the rest were from (MAG). A total of 25% were in the ectomorphic mesomorph category, with (MAG) athletes appearing the most at 16.66%, while the athletes from (WAHS) and (WAG) obtained the same percentage. 18.76% in the mesomorphic ectomorph category, of which 14.58% were athletes from (WAHS), and the rest had the same percentage of athletes from (MAG) and (WAG) 2.09%. Another 12.5% were in the endomorphic ectomorph category, (WAHS) 8.33% and (WAG) 4.17%. 8.33% were located in the balanced mesomorph category, this percentage was 6.25% (WAHS) and the rest (MAG).

In the mesomorph-endomorph category, (WAHS) athletes appeared at 2.08%, another 2.08% were located in the mesomorphic endomorph category (WAHS), and 2.08% of (WAHS) were situated in the balanced ectomorph category.

Table 2 shows the results obtained from the eight movements of athletes in the global flexibility battery test.

**Table 2:** Means and standard deviation of the global flexibility battery test.

Postures	WAHS	WAG	MAG	
	n = 21	n = 15	n = 12	
	Mean(SD)	Mean(SD)	Mean(SD)	*P*
**A MID**	2.10 ± 1.57	1.33 ± 1.11	.42 ± 0.669	.003
**A MIND**	1.76 ± 1.44	0.67 ± 0.724	0.25 ± 0.622	.001
**B MID**	2.95 ± 0.865	2.73 ± 0.594	1.92 ± 0.669	.001
**B MIND**	2.52 ± 1.03	2.13 ± 0.516	1.75 ± 0.622	.035
**C**	2.95 ± 1.39	1.33 ± 0.617	1.25 ± 0.622	.000
**D**	2.71 ± 1.45	2.13 ± 0.915	1.75 ± 0.866	.074
**E MID**	3.05 ± .973	2.20 ± 1.01	1.58 ± .900	.000
**E MIND**	2.29 ± 1.05	1.93 ± .884	1.50 ± .905	.090

The results of the correlations between anthropometric variables, body composition, somatotype, and the global flexibility battery test identified significant positive and negative correlations. Weight was positively correlated with height (r = 0.867; p = 0.000), indicating that weight tended to increase proportionally as height increased. BMI was positively correlated with both weight (r = 0.868; p = 0.000) and height (r = 0.517; p = 0.000), suggesting that BMI tends to increase as weight or height increases. Fat mass also showed positive correlations with weight (r = 0.484; p = 0.000) and BMI (r = 0.606; p = 0.000), indicating that as weight or BMI increased, fat mass in the body also tended to increase. Bone mass was significantly negatively correlated with height (r = -0.289; p = 0.046), weight (r = -0.546; p = 0.000), BMI (r = -0.668; p = 0.000), and fat mass (r = -0.543; p = 0.000), indicating that as height, weight, BMI increase, bone mass tended to be lower. Similarly, muscle mass was negatively correlated with weight (r = -0.378; p = 0.008), BMI (r = -0.479; p = 0.001), and fat mass (r = -0.856; p = 0.000), suggesting that as muscle mass increased, the amount of fat mass tended to decrease. Residual mass was negatively and positively correlated with fat mass (r = -0.507; p = 0.000) and bone mass (r = 0.399; p = 0.005), indicating that residual mass can vary inversely with the amount of body fat and may be positively related to bone mass.

Regarding somatotype variables, significant positive and negative correlations were observed between endomorphy with weight (r = 0.417; p = 0.003), BMI (r = 0.596; p = 0.000), fat mass (r = 0.898; p = 0.000), bone mass (r = -0.602; p = 0.000), residual mass (r = -0.466; p = 0.001), and muscle mass (r = -0.712; p = 0.000). Positive correlations indicate that a higher degree of endomorphy is associated with higher weight, BMI, and fat mass, as opposed to bone mass, residual mass, and muscle mass. Mesomorphy with weight (r = 0.322; p = 0.026), BMI (r = 0.640; p = 0.000), and residual mass (r = -0.466; p = 0.001), indicating that higher mesomorphy was associated with higher weight and BMI and lower residual mass; in contrast, with ectomorphy, significant negative correlations were observed with weight (r = -0.398; p = 0.005), BMI (r = -0.792; p = 0.000), fat mass (r = -0.453; p = 0.001), endomorphy (r = -0.535; p = 0.000), mesomorphy (r = -0.816; p = 0.000), and two positive correlations with bone mass (r = 0.581; p = 0.000), and muscle mass (r = 0.346; p = 0.000). In the X-axis Somatotype, correlations were observed with weight (r = -0.462; p = 0.001), BMI (r = -0.807; p = 0.000), fat mass (r = -0.730; p = 0.000), bone mass (r = 0.671; p = 0.000), muscle mass (r = 0.570; p = 0.000), endomorphy (r = -0.822; p = 0.000). Mesomorphy (r = -0.622; p = 0.000) and with ectomorphy (r = 0.913; p = 0.000). For the Y-axis Somatotype, correlations were observed with BMI (r = 0.599; p = 0.000), residual mass (r = 0.502; p = 0.000), mesomorphy (r = 0.982; p = 0.000), ectomorphy (r = -0.819; p = 0.000), and with the X-axis Somatotype (r = -0.567; p = 0.000).

Additionally, significant positive and negative relationships were observed with the eight postures of the global flexibility battery test. A MID showed a positive relationship with fat mass (r = 0.298; p = 0.040) and a negative one with residual mass (r = -0.422; p = 0.003), while A MIND showed a negative relationship with residual mass (r = -0.372; p = 0.009). B MID showed a negative relationship with residual mass (r = -0.492; p = 0.000), while B MIND showed a negative relationship with residual mass (r = -0.314; p = 0.030). Element C was correlated with fat mass (r = 0.296; p = 0.041) and residual mass (r = -0.352; p = 0.014). Element D showed a relationship with fat mass (r = 0.296; p = 0.041), endomorphy (r = 0.365; p = 0.011), ectomorphy (r = -0.319; p = 0.027), and the X-axis somatotype (r = -0.385; p = 0.007). E MID with bone mass (r = -0.294; p = 0.042) and with residual mass (r = -0.434; p = 0.002). All postures in the the global flexibility battery test presented negative correlations with residual mass. This means that the greater the range of flexibility, the lower the residual mass. This relationship suggests that more flexible athletes have a lower residual mass, which could influence their sports performance.

Positive and negative correlations were identified between anthropometric variables, body composition, somatotypes, and the global flexibility battery test, where the body profile can significantly influence sports performance.

## Discussion

The main objective of this study was to analyze body composition and somatotype concerning the global flexibility battery test in Artistic Gymnastics and Aerial Hoop Sport athletes. These disciplines, which are part of the coordination and competitive art sports (ARC) group, require the comprehensive development of physical and technical abilities to achieve high-perfomance levels [1]

The somatotype, a classification of the human body based on morphology and body composition, is divided into three main categories: ectomorph, mesomorph, and endomorph. Each type has unique characteristics that directly influence athletic ability, ease of gaining muscle or fat, and physical endurance.

**Fig 3:**
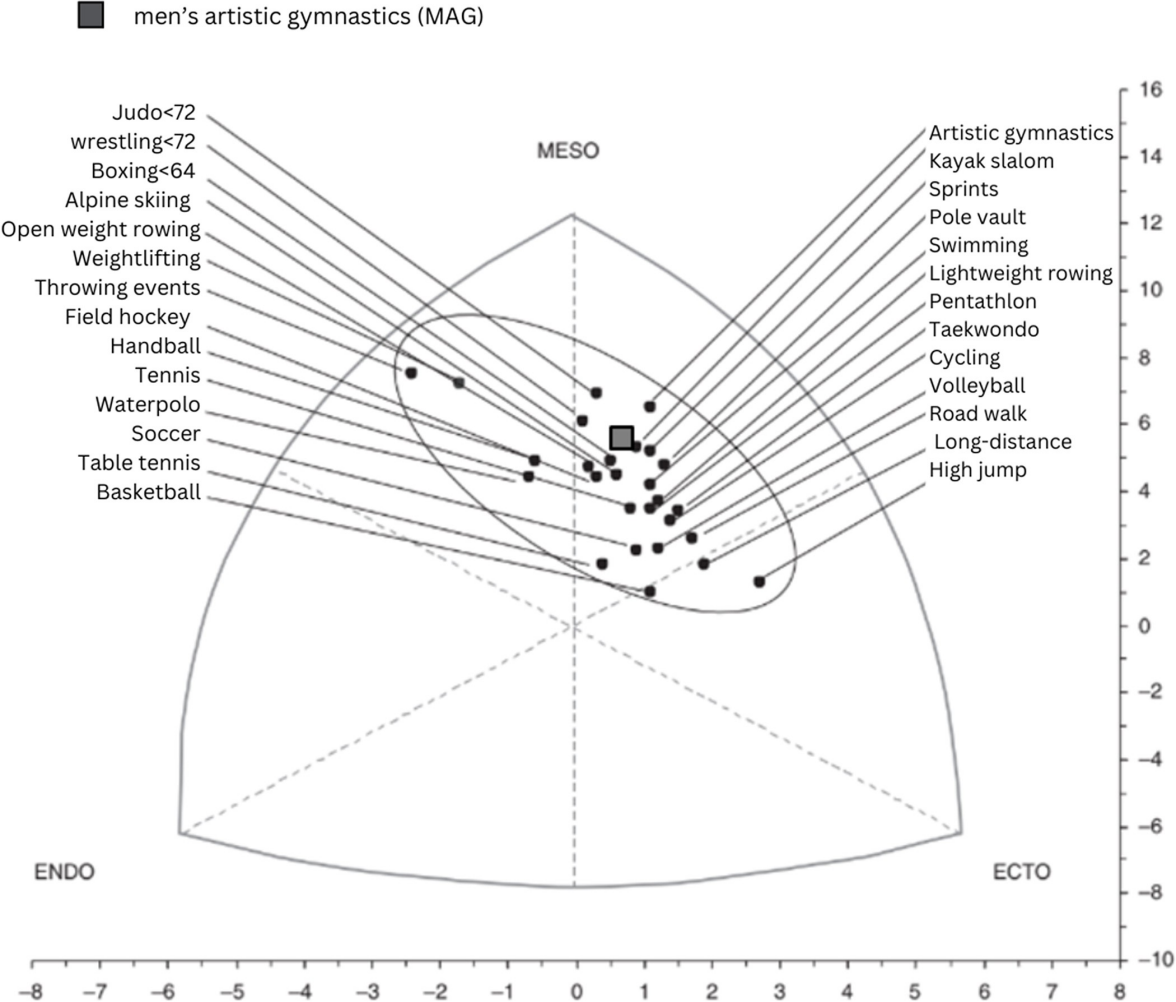
Comparison of the somatopoints of the male branch of various elite sports modified from Pons et al 2015 [8].

In Spain, they have morphologically characterized the sport’s elite by generating a somatocard for males and females, representing each sports discipline [8]. It is important to mention that 12 athletes from the sample are elite athletes (2 MAG, 9 WAHS, 1 WAG), 36 were amateurs, and the dispersion of the amateur athletes occurred along the entire X-axis of the somatotype, showing a similar tendency concerning the mesomorphic component derived from strength training that favors the development of muscle mass, while the elite athletes, in addition to being higher on the Y-axis of the somatotype, which translates into a higher percentage of muscle mass, present slight variation on the X-axis of the somatotype having lower fat mass percentages.

(Fig 3) shows a somatocard, where 2578 men from various sports disciplines at an elite level in Spain were evaluated [8]. The gray box marks the location of the junior athletes from Baja California, México, allowing comparison in the behavior of the data, where it is possible to observe that the somatotype of the gymnasts from Baja California is similar to that of the Spanish athletes with a difference in muscle mass of 45.6% and 48.3%, respectively. It is important to note that of the athletes in this research, only two have an elite competition level.

**Fig 4:**
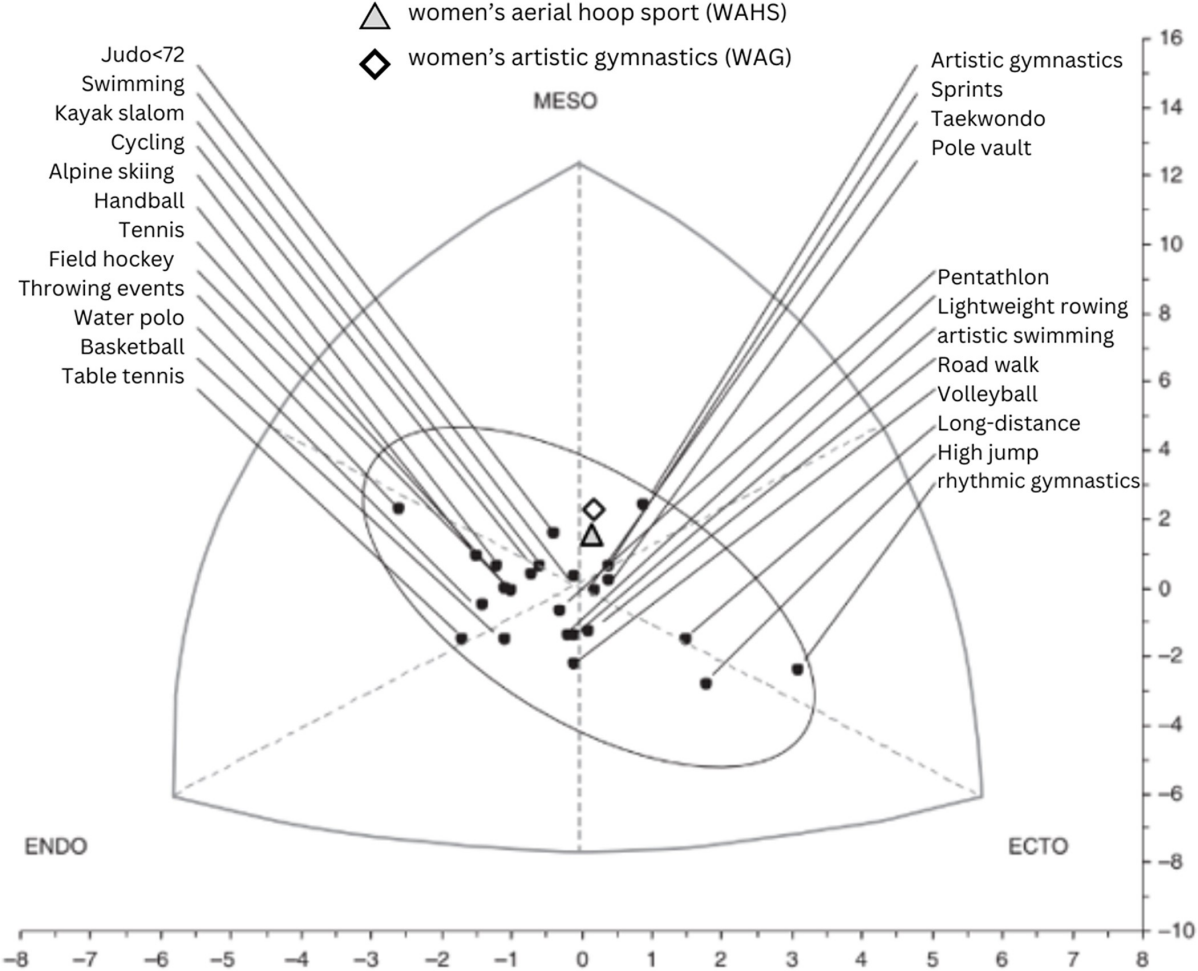
Comparison of the somatopoints of the female branch of various elite sports modified Pons et al 2015 [8].

In (Fig 4), the somatocard of 1491 elite Spanish female athletes from various sports was observed, including the Baja California athletes hoop and gymnastics athletes, represented by a triangle and a diamond, respectively. It was observed that both the hoop athletes and female gymnasts are separate from Spanish athletes. In the group of gymnasts in this study, two-thirds presented a muscular body with an average muscle mass of 44.65%, with differences in the percentage of body fat ranging from 10 to 26%, Wilmore and Costil reported an ideal range of 8 to 16% for this activity [20], while Pons et al report 10% a muscle mass of less than 1 percentage point (45.5 for the Spanish) [8].

When comparing the values obtained from the athletes of aerial hoop sports and women’s artistic gymnastics in this research, a similarity in the percentages of muscle mass was observed with a difference of less than one percentage point. In turn, these data coincide with the values reported for sports such as rhythmic gymnastics, artistic gymnastics, and synchronized swimming from [8], with differences in the % of fat mass, because Spanish athletes are at an elite level and report from 5 to 7 hours of training per day.

Comparing women’s artistic gymnastics and women’s aerial hoop sport in this research, on average, a balanced mesomorphic somatotype is observed with the difference of a more significant muscular component in the case of gymnastics athletes, possibly due to the type of training and competition. For example, in women’s gymnastics during the vaulting test, a short run is followed by a jump with a rebound on the board and the subsequent rotation of the body on the transverse and longitudinal axes, or both simultaneously, ending in a precise landing. This test has a duration between 6 and 8 s, which indicates that explosive strength associated with type II b muscle fibers (fast-twitch) is required, which is associated with a larger diameter than slow-twitch fibers and, therefore, greater muscle mass (mesomorphic component). In their competition routine, aerial hoop athletes reach 3 min and 50 s. Therefore, their muscles must be capable of withstanding the fatigue caused by prolonged contractions, which translates into type I and type II fibers of smaller muscle volume.

This coincides with research on somatotype [7, 9, 21], where it was concluded that elite athletes often present a standard profile that fits the needs of their sport [22]. In this regard, aerial hoop athletes, with a higher technical level have a somatotype that coincides with that presented in (Fig 4), where they are located near competitive art sports, rhythmic gymnastics, artistic gymnastics, and synchronized swimming. Therefore, data on body composition and somatotype are key factors concerning sports performance [23] as well as being very useful for coaches, as they allow for a clear goal regarding the ideal somatotype for this newly created sport, for which there are no reference data in the current literature.

## Conclusions

The study results indicate a significant relationship between athlete’s somatotypes, body composition, and flexibility test performance. Significant differences were identified between the athletes of men’s Artistic Gymnastics (MAG), women’s Aerial Hoop Sport (WAHS), and women’s Artistic Gymnastics (WAG) in terms of fat mass, bone mass, residual mass, and the proportions of endomorphy, mesomorphy, and ectomorphy. These differences suggest that somatotype may be a factor in sports performance, as correlations were found between anthropometric and body composition measurements and the global flexibility battery test.

On the other hand, greater flexibility is related to a lower residual mass, which could impact the agility and efficiency of the athletes’ movements.

In conclusion, athletes’ somatotype profiles appear to significantly impact their body composition and flexibility, which in turn may affect their sports performance. These findings highlight the importance of considering somatotypes in assessing and training athletes to optimize their preparation and performance in their respective disciplines.
